# Microwave-assisted synthesis and antioxidant properties of hydrazinyl thiazolyl coumarin derivatives

**DOI:** 10.1186/1752-153X-6-32

**Published:** 2012-04-17

**Authors:** Hasnah Osman, Afsheen Arshad, Chan Kit Lam, Mark C Bagley

**Affiliations:** 1 School of Chemical Sciences, Universiti Sains Malaysia, 11800, Penang, Malaysia; 2 School of Pharmaceutical Sciences, Universiti Sains Malaysia, 11800, Penang, Malaysia; 3 Department of Chemistry, School of Life Sciences, University of Sussex, Brighton, BN1 9QJ, UK

**Keywords:** Coumarins, Thiazoles, Antioxidant activity, Microwave synthesis

## Abstract

**Background:**

Coumarin derivatives exhibit a wide range of biological properties including promising antioxidant activity. Furthermore, microwave-assisted organic synthesis has delivered rapid routes to *N*- and *O*-containing heterocycles, including coumarins and thiazoles. Combining these features, the use of microwave-assisted processes will provide rapid access to a targeted coumarin library bearing a hydrazino pharmacophore for evaluation of antioxidant properties

**Results:**

Microwave irradiation promoted 3 of the 4 steps in a rapid, convergent synthesis of a small library of hydrazinyl thiazolyl coumarin derivatives, all of which exhibited significant antioxidant activity comparable to that of the natural antioxidant quercetin, as established by DPPH and ABTS radical assays

**Conclusions:**

Microwave dielectric heating provides a rapid and expedient route to a series of hydrazinyl thiazolyl coumarins to investigate their radical scavenging properties. Given their favourable properties, in comparison with known antioxidants, these coumarin derivatives are promising leads for further development and optimization.

## Background

The synthesis and biological activities of coumarin derivatives occupy an important position in heterocyclic chemistry as well as in medicinal chemistry. The compounds containing this heterocyclic motif are widely found as additives in food, in cosmetic products, as pharmaceutical agents [1] and as luminescent materials [2]. They have pronounced medicinal value as anticoagulants [3], free radical scavengers [4,5], and as lipoxygenase [6] and cyclooxygenase inhibitors [7]. Moreover, many coumarins exhibit high antibacterial [8], antifungal [9] and cytotoxic activities [10]. The incorporation of a 3-thiazolyl substituent can further enhance the activity of this pharmacophore: thiazolyl coumarins have been reported to exhibit anticonvulsant [11], anticancer, antimicrobial [12], analgesic and anti-inflammatory properties [13] and display good activity against *Mycobacterium tuberculosis*[14] and *Helicobactor pylori*[15]. The pathophysiology of many of the above-mentioned diseases, and others, has been linked with oxidative stress, produced in our body as a result of various oxidation processes essential for life. Although the importance of antioxidants to prevent the progression of age-related diseases, or interfere in the ageing process itself, could be contested [16,17], their role in enzymatic and non-enzymatic defense mechanisms in both the lipid and aqueous phase is well established [18]. Given that coumarin and its derivatives are well-known as antioxidants [19], we set out to access a series of thiazolyl coumarin derivatives, rapidly and in a convergent manner, to explore their antioxidant properties. Previously we have reported the synthesis and crystalline structure of various thiazolyl coumarin derivatives prepared by conventional methods, including the synthesis of a series of hydrazinyl thiazolyl coumarins [20-24]. Given the versatility and capability of microwave-assisted synthesis for rapid delivery of compounds of biological interest [25-28], our previous success in the use of microwave irradiation for the rapid synthesis of thiazoles of biological interest [29,30], and the previous application of this technology in the synthesis of coumarins, both on small and large scale [31-34], as well as thiazolyl coumarin Schiff bases [35], we set out to investigate a rapid microwave-assisted synthesis of a hydrazinyl thiazolyl coumarin library for evaluation of in vitro antioxidant activity by DPPH radical and ABTS radical cation assays, using the natural antioxidant quercetin and a synthetic antioxidant (butylatedhydroxytoluene) as reference standards.

## Results and discussion

**Scheme 1:**
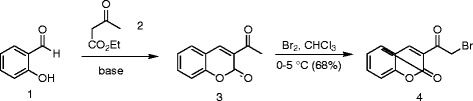
Synthesis of 3-(bromoacetyl)coumarin (4) component for Hantzsch thiazole synthesis.

3-(Bromoacetyl)coumarin (**4**), a key precursor of Hantzsch thiazole synthesis for the hydrazinyl thiazolyl coumarin library, was prepared by a two-step sequence (Scheme 1). Firstly Knoevenagel condensation, with spontaneous α-pyrone **3** formation, was investigated by heterocyclocondensation of salicylaldehyde (**1**) and ethyl acetoacetate (**2**) under microwave irradiation using a range of conditions (Table1). It has been reported that the use of microwave heating is beneficial for 3-acetylcoumarin synthesis, allowing for low catalyst loadings and short reaction times to limit the generation of unwanted side products [33,34]. Although solvent-free conditions have been used for α-pyrone **3** formation under microwave irradiation [31,32] and conventional heating [36], in the presence of a catalytic amount of piperidine or l-proline, these give rise to widely varying reaction times, operating temperatures and thus chemical yields [31]. In our hands (entry 1), the solvent-free process resulted in a rapid rise of pressure and so was discarded in favour of the more reliable Leadbeater method, carried out in ethanol solvent, which has been reported to proceed in reasonable yield (67–81%) on multigram scale in sealed vessel microwave apparatus [33] and more recently in a large scale batch reactor [34]. In our modified procedure, on 18 mmol scale, with a small excess of carbonyl compound **2** (1.3 equiv), the use of piperidine base (entries 6–8) seemed superior to the l-proline catalyst (entries 2–5) giving 3-acetylcoumarin (**3**) in 99% isolated yield in a reaction time of only 5 min (entry 8) following purification by recrystallization.

**Table 1:** Conditions for the microwave-assisted synthesis of 3-acetylcoumarin (3)

**Entry**	**Base**	**Reagents & conditions**^**†**^	**Yield%**^**‡**^
1	Piperidine	Solvent-free, 50°C, 5 min	-^*a*^
2	l-Proline	EtOH, 50°C, 5 min	54
3	l-Proline	EtOH, 50°C, 10 min	59
4	l-Proline	EtOH, 120°C, 1 min	72
5	l-Proline	EtOH, 120°C, 2 min	93
6	Piperidine	EtOH, 120°C, 1 min	74
7	Piperidine	EtOH, 120°C, 10 min	98
8	Piperidine	EtOH, 50°C (20 W), 5 min	99

^†^ All reactions were carried out using 5 mol% of base under microwave dielectric heating at the given temperature in a sealed tube using a CEM Discover single-mode microwave synthesizer by moderation of the initial magnetron power (20–150 W). ^‡^ Isolated yield after recrystallization (ethanol). ^*a*^ Reaction was halted upon the rapid build-up of pressure.

**Scheme 2:**
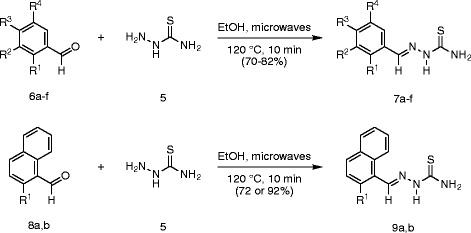
Synthesis of the thiosemicarbazone (7 or 9) component for Hantzsch thiazole synthesis.

With a rapid and highly efficient route to acetylcoumarin **3** established, the first of the building blocks for Hantzsch thiazole synthesis, bromoacetylcoumarin **4**, was prepared in 68% yield by the electrophilic bromination of acetylcoumarin **3**, in CHCl_3_, according to the method of Gursoy and Karali [14] (Scheme 1). Furthermore the second, thioamide, component was provided by the condensation of thiosemicarbazide **5** and one member of a subset of benzaldehydes **6** or naphthaldehydes **8** in a microwave-assisted condensation. Microwave irradiation of a methanolic solution of a range of these precursors at 120°C for 10 min (Scheme 2) gave thiosemicabazones **7** or **9**, respectively, in excellent isolated yield (70–92%) after purification by recrystallization (Table2). The efficiency of the microwave-assisted procedure compared highly favourably, in terms of isolated yield and reaction time, with the condensation carried out using more traditional methods, at reflux in MeOH in the presence of AcOH for 1–4 h, using the same isolation and purification regime.

**Table 2:** Isolated yields for the microwave-assisted condensation of benzaldehydes 6 or naphthaldehydes 8 with thiosemicarbazide 5 and comparison with traditional methods

**Entry**	**Compound**	**R**^1^	**R**^2^	**R**^3^	**R**^4^	**Yield% under microwave irradiation**^†^	**Yield% using traditional conditions**^‡^
1	**7a**	OH	H	H	H	70	69
2	**7b**	H	OH	H	H	82	66
3	**7c**	H	H	OH	H	71	71
4	**7d**	OH	H	OH	H	*n/a*	76
5	**7e**	OH	H	H	Br	78	64
6	**7f**	OH	OMe	H	H	72	69
7	**9a**	H	-	-	-	92	67
8	**9b**	OH	-	-	-	72	62

^† ^Isolated yield after microwave dielectric heating in EtOH at 120°C for 10 min (hold time) in a sealed tube using a CEM Discover single-mode microwave synthesizer by moderation of the initial magnetron power (20–150 W), followed by purification by recrystallization (EtOH). ^‡ ^Isolated yield after reaction by traditional conductive heating at reflux in MeOH in the presence of AcOH for 1–4 h and purification by recrystallization (EtOH or EtOH–CHCl_3_) (see reference 20 for details). *n/a* Experiment not investigated.

**Scheme 3:**
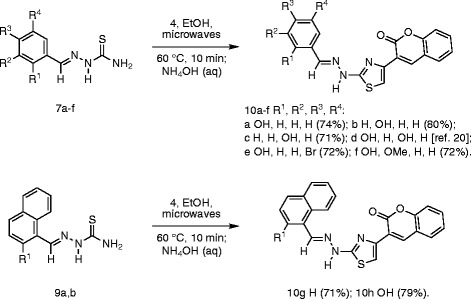
Hantzsch synthesis of thiazolyl coumarins 10a-h.

Given that microwave irradiation has been used before in the synthesis of thiazole derivatives of biological interest [29,30,37-39] and that, in particular, Hantzsch thiazole synthesis conducted in ethanol was successful in generating thiazoles in good yields for evaluation of antiproliferative activity [40], the Hantzsch synthesis of thiazolyl coumarins **10a-h** from corresponding building blocks was investigated under related conditions (Scheme 3). Microwave irradiation of semicarbazone **79** and bromoketone **4** in ethanol at 60°C for 10 min (hold time) followed by treatment with ammonium hydroxide (5%) gave coumarin derivatives **10a-h** in very good yield (71–80%) (Table3). In all cases, the isolated yield after purification by recrystallization was closely comparable with traditional conductive heating methods (see Table3). The identity and purity was confirmed by analysis of spectroscopic and mass spectrometric data and by comparing the melting point with literature values [20]. The use of microwave irradiation had facilitated and accelerated 3 out of the 4 steps in the convergent synthesis of thiazolyl coumarins **10a-h**, including two heterocyclocondensations (α-pyrone formation and Hantzsch thiazole synthesis) and semicabazone formation, to provide an extremely rapid route to a focused library for examination of antioxidant properties.

**Table 3:** Comparison of isolated yields for microwave-assisted and conventional Hantzsch synthesis of thiazolyl coumarins 10

**Entry**	**Compound**	**Yield% under microwave irradiation**^**†**^	**Yield% using traditional conditions**^**‡**^
1	**10a**	74	70
2	**10b**	80	72
3	**10c**	71	80
4	**10d**	*n/a*	72
5	**10e**	72	78
6	**10f**	72	70
7	**10g**	71	70
8	**10h**	79	75

^† ^Isolated yield after microwave dielectric heating in EtOH at 60°C for 10 min (hold time) in a sealed tube using a CEM Discover single-mode microwave synthesizer by moderation of the initial magnetron power (30 W), followed by treatment with aqueous base and purification by recrystallization. ^‡ ^Isolated yield after reaction by traditional conductive heating at reflux in EtOH–CHCl_3 _for 1–4 h, basification with NH_3 _(aq) and purification by recrystallization (see reference 20 for details). *n/a* Experiment not investigated.

**Figure 1:**
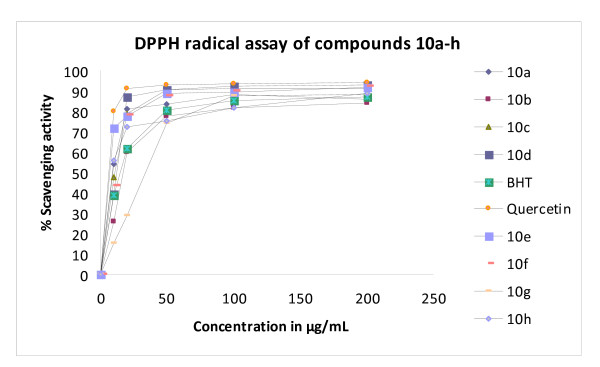
Evaluation of antioxidant properties by DPPH assay.

**Figure 2:**
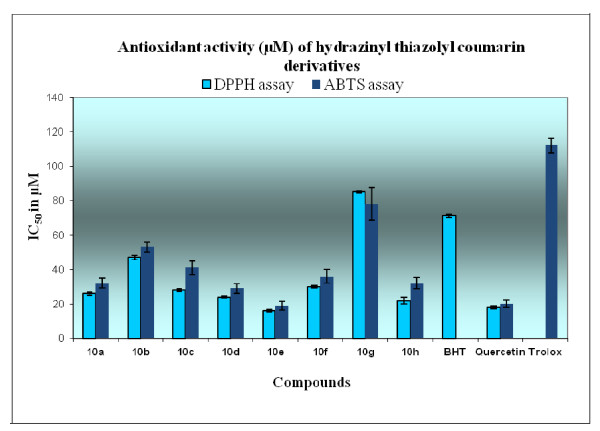
Comparing antioxidant activities using DPPH and ABTS assays.

**Scheme 4:**
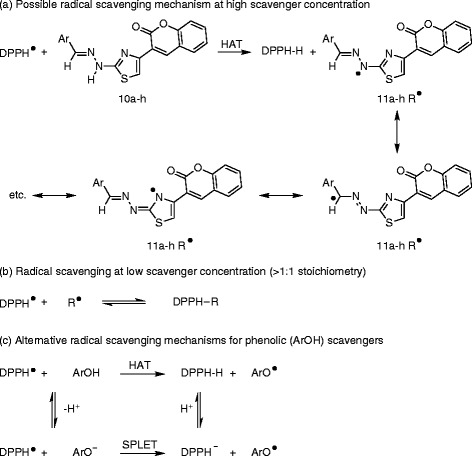
**DPPH radical scavenging reactions of hydrazinyl thiazolyl coumarins 10a-h and phenolic (ArOH) scavengers **[41,42]**.**

Hydrazino-thiazole derivatives have been shown recently to possess radical scavenging ability and some simple structure-activity relationships have been described for a small library of compounds [41]. The antioxidant properties of this motif were evaluated quickly and efficiently using a 1,1-diphenyl-2-picrylhydrazyl (DPPH) radical scavenging model [42] in which the decrease in the strong absorption band at *ν* 517 nm due to the unpaired electron in DPPH decreased stoichiometrically on scavenging an electron or hydrogen atom. Using the DPPH free radical assay, according to a modification of our previously reported procedure [43], the radical scavenging activity of this new series of thiazolyl coumarins **10a-h** was determined and expressed as IC_50_ values [41,43], which is the concentration of tested compound required to scavenge 50% of the DPPH radical concentration (0.11 mM in this case on dilution). All of the DPPH assays were conducted in triplicate and both a synthetic antioxidant, 2,6-di-*tert*-butyl-4-methylphenol (BHT), and the natural antioxidant quercetin were used as reference standards. The results from the DPPH radical scavenging assay were validated by 2,2′-azinobis(3-ethylbenzothiazoline-6-sulfonate) (ABTS) assay [42,44], which established IC_50_ values for each tested compound to trap the ABTS^·+^ radical. For consistency, IC_50_ values in ABTS assay were established in triplicate and compared with quercetin, again, and Trolox, which is a common reference antioxidant for interaction with ABTS^·+^[42,44-46]. As expected, DPPH radical assay indicated that radical scavenging activity was dose dependent and increased with the concentration of the tested compound (Figure1). The IC_50_ values of thiazolyl coumarins **10a-h** in the DPPH assay were found to be in the range 16–85 μM (Table4), displaying excellent antioxidant activity above and beyond that of BHT and comparable to that of quercetin (Figure2). It was worthy to note that compounds **10a**, **10c**, **10d**, **10e, 10f** and **10h** exhibited very high activity against the DPPH radical (at a concentration of 0.11 mM) with IC_50_ values in the order of 16–30 μM. The activity of this thiazolyl coumarin library compared closely with other known hydrazino-thiazoles (IC_50_ 15–60 μM at a DPPH radical concentration of 0.1 mM) [41] implying that the coumarin and phenolic functions had not adversely affected antioxidant activity. The activity of these compounds was attributed for the most part to the hydrazinothiazole functionality but clearly was modulated and enhanced by the incorporation of other unique groups. The presence of the hydrazino N-H group (Scheme 4) and the phenolic hydroxyl were viewed as important structural features, both having the ability for hydrogen atom transfer (HAT) to the DPPH free radical to give a resonance stabilized radical **11a-f**. The mesomeric stabilization of this radical, in particular through the addition of electron donating aromatic units could contribute to radical scavenging ability, with notable improvements observed for 2-hydroxy **10a** and 4-hydroxyphenyl **10c** analogues over the 3-hydroxy precursor **10b** (Table4, entry 2). Alternatively sequential proton loss electron transfer (SPLET) could compete with HAT (Scheme 4c) for phenolic scavengers (ArOH) as has been described for curcumin [42]. In this latter case, synergistic contributions to the activity of phenolic and hydrazino scavengers could not be ruled out but these features were not explored further. The presence of hydroxyl groups in compounds **10a-10f** and **10h** appeared to contribute to the antioxidant activity of these compounds and when absent resulted in a dramatic loss of radical scavenging ability (Table4, compare lower activity of **10g** in entry 7 with entry 8), suggesting the involvement of a SPLET mechanism. The reaction of the DPPH radical with scavengers (Scheme 4a) in general suggests a 1:1 stoichiometry of reaction at high scavenger concentration. When the concentration of the tested compounds is significantly lower than the DPPH radical, as could be the case for compounds with potent IC_50_ values, the remaining DPPH radical may combine with the hydrazinyl thiazolyl radical **11a-f** (Scheme 4b) and thus, the stoichiometry of the reaction could appear higher than 1:1 [41]. Finally, on review it was bromophenyl analogue **10e** that exhibited the highest activity in the DPPH assay (Table4, entry 5) even exceeding the activity of the natural antioxidant quercetin (entry 10). 

**Table 4:** IC_50_ for DPPH and ABTS radical scavenging ability of hydrazinyl thiazolyl coumarins **10** and comparison with known antioxidants BHT and quercetin

**Entry**	**Compound**	**R**^**1**^	**R**^**2**^	**R**^**3**^	**R**^**4**^	**DPPH assay IC**_**50**_**/μM**^**†**^	**ABTS assay IC**_**50**_**/μM**^**‡**^
1	**10a**	OH	H	H	H	26 ± 0.96	32 ± 3.06
2	**10b**	H	OH	H	H	47 ± 1.24	53 ± 2.74
3	**10c**	H	H	OH	H	28 ± 0.62	41 ± 3.95
4	**10d**	OH	H	OH	H	24 ± 0.67	29 ± 1.88
5	**10e**	OH	H	H	Br	16 ± 0.82	19 ± 2.76
6	**10f**	OH	OMe	H	H	30 ± 0.78	36 ± 3.18
7	**10g**	H	-	-	-	85 ± 0.66	78 ± 5.64
8	**10h**	OH	-	-	-	22 ± 1.75	32 ± 3.42
9	BHT	-	-	-	-	71 ± 0.82	-
10	quercetin	-	-	-	-	18 ± 0.99	20 ± 2.19
11	Trolox	-	-	-	-	-	112 ± 4.29

^†^ Required concentration of the tested compound to scavenge 50% of the DPPH radical present at a concentration of 0.1 mM; average from 3 assays. ^‡^ Required concentration of the tested compound to scavenge 50% of the ABTS radical present at a concentration of 0.12 mM; average from 3 assays.

The findings from the DPPH assays correlated well with IC_50_ values derived in ABTS experiments. The ABTS radical was generated by treatment with the strong oxidizing agent K_2_S_2_O_8_ and then reduced by the addition of the antioxidant; this was observed by the suppression of the characteristic long wave absorption of ABTS^·+^. For the most part, the mean IC_50_ values were of very similar magnitude with minimal differences between the ABTS and DPPH values (Table4), an observation that has been made before in measuring antioxidant activities of sorghum products [47]. Thus no matter which assay was used, the compounds exhibited the same activity trends and SAR (Figure2), validating the reliability of both techniques. However, it was noted that there appeared to be greater consistency with the DPPH data and greater variability in the ABTS IC_50_ values according to the observed standard deviations.

## Conclusions

The synthesis and antioxidant activity of hydrazinyl thiazolyl coumarin derivatives have been reported. Microwave irradiation promoted 3 out of the 4 steps to establish an extremely rapid, convergent and highly efficient route to the target library. All of the compounds were purified by recrystallization and obtained in good isolated yields, with spectroscopic and physical data that fully supported the proposed structures. DPPH and ABTS assays indicated that almost all of the synthesized hydrazinyl thiazolyl coumarin derivatives (**10a**, **10c**, **10d**, **10e**, **10f** and **10h**) had significant radical scavenging activity that was comparable or better than the known antioxidants, quercetin, BHT and Trolox. The thiazole, hydrazino, and phenolic moieties are the likely structural components contributing to free radical scavenging activity. However, the facile incorporation of the coumarin motif offers an opportunity to further optimize radical scavenging activity in the future for hit-to-lead development of new and improved synthetic antioxidants.

## Experimental

### Preparation of 3-acetyl-*2H*-chromen-2-one (3) by knoevenagel condensation

Piperidine (0.1 mL) was added dropwise to a mixture of salicylaldehyde (2.0 mL, 18 mmol) and ethyl acetoacetate (3.0 mL, 24 mmol) in EtOH (1.0 mL) The reaction mixture was stirred under microwave irradiation for 5 min (hold time) at 50°C (initial power 20 W) and then was cooled in a stream of compressed air, resulting in a yellow solid. Purification by recrystallization (EtOH) gave the *title compound****3*** (3.3 g, 99%) as fine yellow needles, mp 118–119°C (Lit. mp 119–121°C [48]); IR (KBr) ν_max_ 2930 (C-H aliphatic), 1742 (C = O), 1677 (O-C = O); ^1^H NMR (500 MHz, DMSO-*d*_*6*_) δ 8.58 (1H, s), 7.89 (1H, dd, *J* = 7.6, 1.6 Hz), 7.70 (1H, ddd, *J* = 8.1, 7.6, 1.6 Hz), 7.40 (1H, d, *J* = 8.1 Hz), 7.38 (1H, td, *J* =7.6, 1 Hz), 2.55 (3H, s).

Alternatively l-proline (0.18 g, 16 mmol) was added to a mixture of salicylaldehyde (2.0 mL, 18 mmol) and ethyl acetoacetate (3.0 mL, 24 mmol) in EtOH (1.0 mL). The reaction mixture was stirred under microwave radiation for 1–10 min (hold time) at 50 or 120°C, resulting in the formation of a yellow solid. Purification by recrystallization (EtOH) gave the title compound **3** (2.8 g, 93%) (Table2, entry 4) as fine yellow needles, mp 117–118°C (Lit. mp 119–121°C [48]) with identical spectroscopic properties.

### Bromination of acetylcoumarins

3-Acetyl-*2H*-chromen-2-one (**3**) (20 g, 0.11 mol), obtained from the combined product of a series of parallel experiments, was dissolved in alcohol-free CHCl_3_ (20 mL) and a solution of Br_2_ (5.45 mL, 0.11 mol) in CHCl_3_ (20 mL) was added dropwise from a dropping funnel with constant stirring at 0–5°C. After 3 h, a dark yellow solid separated. The reaction mixture was heated for 15 min at reflux, cooled and CHCl_3_ was removed using a rotary evaporator. Purification by recrystallization (glacial AcOH) gave 3-bromoacetylcoumarin **4** (19 g, 68%) as off-white needles, mp 162–165°C (Lit. mp 160–163°C [11]); IR (KBr) ν_max_ 2930 (C-H aliphatic), 1731 (C = O), 1686 (O-C = O); ^1^H NMR (400 MHz, CDCl_3_) δ 8.66 (1H, s), 7.69–7.75 (2H, m), 7.39–7.44 (2H, m), 4.79 (2H, s).

### General procedure for the preparation of thiosemicarbazones 7a-f or 9a,b from aldehydes 6a-f or 8a,b, respectively

Thiosemicarbazide (**5**) (0.46 g, 5.00 mmol) was added slowly to a stirred solution of salicylaldehyde (0.11 mL, 5.00 mmol) in hot absolute EtOH (5 mL). The resulting solution was heated at 120°C for 10 min (hold time) under microwave irradiation. The mixture was cooled in a stream of compressed air, then allowed to cool further in an ice bath for 30 min to give a colourless precipitate, which was filtered and washed with cold water. Purification by recrystallization (95% EtOH) gave (*E*)*-*2-(2-hydroxybenzylidene)hydrazinecarbothioamide (**7a**). A similar procedure was employed for the preparation of the other 2-(benzylidene)hydrazinecarbothioamides **7b-f**.

***(*E*)-2-(2-Hydroxybenzylidene)hydrazinecarbothioamide (7a)*** (0.68 g, 70%) was obtained as colourless crystals, mp 208–210°C (Lit. mp 210°C [49]); IR (KBr) ν_max_ 3462, 3415 (NH_2_), 3374 (OH), 3215 (NH), 1610 (C = N), 1230 (C = S); with identical spectroscopic properties. ***(*E*)-2-(3-Hydroxybenzylidene)hydrazinecarbothioamide (7b)*** (0.80 g, 82%) was obtained as light brown crystals, mp 166–168°C (Lit. mp 168–170°C [50]); IR (KBr) ν_max_ 3458, 3409 (NH_2_), 3365 (OH), 3260 (NH), 1614 (C = N), 1225 (C = S); with identical spectroscopic properties. ***(*E*)-2-(4-Hydroxybenzylidene)hydrazinecarbothioamide (7c)*** (0.69 g, 71%) was obtained as colourless crystals, mp 214–216°C (Lit. mp 218–219°C [46]); IR (KBr) ν_max_ 3469, 3414 (NH_2_), 3377 (OH), 3235 (NH), 1610 (C = N), 1231 (C = S); with identical spectroscopic properties. ***(*E*)-2-(5-Bromo-2-hydroxybenzylidene)hydrazinecarbothioamide (7e)*** (1.10 g, 78%) was obtained as a beige solid, mp 234–236°C (Lit. mp 238.5°C [51]); IR (KBr) ν_max_ 3543, 3429 (NH_2_), 3317 (OH), 3253 (NH), 1612 (C = N), 1217 (C = S); with identical spectroscopic properties. ***(*E*)-2-(2-Hydroxy-3-methoxybenzylidene)hydrazinecarbothioamide (7f)*** (0.80 g, 72%) was obtained as a colourless solid, mp 222–224°C (Lit. mp 220–222°C [52]); IR (KBr) ν_max_ 3458, 3424 (NH_2_), 3342 (OH), 3164 (NH), 1594 (C = N), 1140 (C = S); with identical spectroscopic properties.

### General procedure for the synthesis of 2-(naphthalen-1-ylmethylene)hydrazinecarbothioamides 9

The experimental procedure employed for the synthesis of (*E*)-2-(2-hydroxybenzylidene)hydrazinecarbothioamide (**7a**) was employed using naphthaldehyde **8** (5.0 mmol), thiosemicarbazide (**5**) (5.0 mmol) and absolute EtOH. Purification by recrystallization, using EtOH–EtOAc (1:2), gave the target compound (**9a,b**). ***(*E*)-2-(Naphthalen-1-ylmethylene)hydrazinecarbothioamide (9a)*** (1.05 g, 92%) was obtained as a yellow solid, mp 138–140°C (Lit. mp 126°C [53]); IR (KBr) ν_max_ 3448, 3412 (NH_2_), 3228 (NH), 1592 (C = N), 1249 (C = S); with identical spectroscopic properties. ***(*E*)-2-[(2-Hydroxynaphthalen-1-yl)methylene]hydrazinecarbothioamide (9b)*** (0.88 g, 72%) was obtained as a pink-yellow solid, mp 272–274°C (Lit. mp 271°C [54]); IR (KBr) ν_max_ 3550, 3426 (NH_2_), 3236 (NH), 3148 (OH), 1589 (C = N), 1226 (C = S); with identical spectroscopic properties.

### Synthesis of hydrazinyl thiazolyl coumarin derivatives 10a-f from bezylidenethiosemicabazides 7a-f

A stirred solution of 3-bromoacetylcoumarin (**4**) (107 mg, 0.40 mmol) and benzylidenethiosemicarbazone **7a-f** (78 mg, 0.40 mmol) in EtOH was heated at 60°C for 10 min (hold time) under microwave irradiation and then cooled in a stream of compressed air to give a thick yellow precipitate. The reaction mixture was neutralized with aqueous ammonium hydroxide solution (5%) and the precipitated solid was filtered. Purification by recrystallization, using CHCl_3_–EtOH (1:3), gave (*E*)-3-(2-(2-(2-hydroxybenzylidene)hydrazinyl)thiazol-4-yl)-*2H*-chromen-2-one (**10a**). A similar procedure was employed to prepare the target compounds **10b-h**, after purification by recrystallization using CHCl_3_, EtOH or EtOAc–EtOH.

***(*E*)-3-{2-[2-(2-Hydroxybenzylidene)hydrazinyl]thiazol-4-yl}-2*H*-chromen-2-one (10a)***

The title compound (**10a**) (107 mg, 74%) was obtained after purification by recrystallization, using CHCl_3_–EtOH (1:3), as a yellow solid, mp 270–272°C (Lit. mp 270–272°C [20]); IR (KBr) ν_max_ 3420 (NH), 3212 (OH), 1699 (O-C = O), 1603 (C = N); ^1^H NMR (400 MHz, DMSO-*d*_*6*_) δ 12.30 (1H, br s), 10.41 (1H, br s), 8.55 (1H, s), 8.29 (1H, s), 7.87 (1H, dd, *J* = 7.5, 1.2 Hz), 7.77 (1H, s), 7.65 (1H, dd, *J* = 8.0, 1.1 Hz), 7.64 (1H, ddd, *J* = 8.2, 7.5, 1.2 Hz), 7.47 (1H, d, *J* = 8.2 Hz), 7.40 (1H, t, *J* = 7.5 Hz), 7.37 (1H, td, *J* = 8.0, 1.1 Hz), 6.91 (1H, d, *J* = 8.0 Hz), 6.89 (1H, t, *J* = 8.0 Hz).

***(*E*)-3-{2-[2-(3-Hydroxybenzylidene)hydrazinyl]thiazol-4-yl}-2*H*-chromen-2-one (10b)***

The title compound (**10b**) (116 mg, 80%) was obtained after purification by recrystallization, using EtOAc–EtOH (1:2), as bright yellow crystals, mp 253–254°C (Lit. mp 254–256°C [20]); IR (KBr) ν_max_ 3365 (NH), 3262 (OH), 1698 (O-C = O), 1602 (C = N); ^1^H NMR (400 MHz, DMSO-*d*_*6*_) δ 12.18 (1H, br s), 9.63 (1H, br s), 8.55 (1H, s), 7.98 (1H, s), 7.87 (1H, dd, *J* = 7.6, 0.9 Hz), 7.78 (1H, s), 7.64 (1H, ddd, *J* = 8.2, 7.6, 0.9 Hz), 7.46 (1H, d, *J* = 8.2 Hz), 7.40 (1H, t, *J* = 7.6 Hz), 7.23 (1H, t, *J* = 7.8 Hz), 7.12 (1H, s), 7.05 (1H, d, *J* = 7.8 Hz), 6.80 (1H, dd, *J* = 7.8, 2.4 Hz).

***(*E*)-3-{2-[2-(4-Hydroxybenzylidene)hydrazinyl]thiazol-4-yl}-2*H*-chromen-2-one (10c)***

The title compound (**10c**) (103 mg, 71%) was obtained after purification by recrystallization, using CHCl_3_–EtOH (1:3), as brown crystals, mp: 249–251°C (Lit. mp 249–250°C [20]); IR (KBr) ν_max_ 3424 (NH), 3212 (OH), 1706 (O-C = O), 1605 (C = N); ^1^H NMR (400 MHz, DMSO-*d*_*6*_) δ 12.05 (1H, br s), 9.93 (1H, br s), 8.60 (1H, s), 7.93 (1H, s), 7.81 (1H, dd, *J* = 7.6, 1.0 Hz), 7.72 (1H, s), 7.67 (1H, ddd, *J* = 8.2, 7.6, 1.0 Hz), 7.55 (2H, d, *J* = 8.6 Hz), 7.48 (1H, d, *J* = 8.2 Hz), 7.44 (1H, t, *J* = 7.6 Hz), 6.88 (2H, d, *J* = 8.6 Hz).

***(*E*)-3-{2-[2-(2-Hydroxy-5-bromobenzylidene)hydrazinyl]thiazol-4-yl)-2*H*-chromen-2-one (10e)***

The title compound (**10e**) (127 mg, 72%) was obtained after purification by recrystallization, using EtOAc–EtOH (3:1), as a yellow solid, mp: 295–296°C (Lit. mp 296–298°C [20]); IR (KBr) ν_max_ 3430 (NH), 3260 (OH), 1701 (O-C = O), 1583 (C = N); ^1^H NMR (400 MHz, DMSO-*d*_*6*_) δ 12.30 (1H, br s), 10.45 (1H, br s), 8.57 (1H, s), 8.32 (1H, s), 7.88 (1H, dd, *J* = 7.5, 1.1 Hz), 7.81 (1H, s), 7.79 (1H, d, *J* = 2.5 Hz), 7.68 (1H, ddd, *J* = 8.3, 7.5, 1.1 Hz), 7.50 (1H, d, *J* = 8.3 Hz), 7.44 (1H, t, *J* = 7.5 Hz), 7.38 (1H, dd, *J* = 8.5, 2.5 Hz), 6.87 (1H, d, *J* = 8.5 Hz).

***(*E*)-3-{2-[2-(2-Hydroxy-3-methoxybenzylidene)hydrazinyl]thiazol-4-yl}-2*H*-chromen-2-one (10f)***

The title compound (**10f**) (113 mg, 72%) was obtained after purification by recrystallization, using EtOAc–EtOH (2:1), as a brown solid, mp 266–267°C (Lit. mp 266°C [20]); IR (KBr) ν_max_ 3445 (NH), 3239 (OH), 1704 (O-C = O), 1600 (C = N); ^1^H NMR (400 MHz, DMSO-*d*_*6*_) δ 12.18 (1H, br s), 9.45 (1H, br s), 8.56 (1H, s), 8.39 (1H, s), 7.87 (1H, dd, *J* = 7.5, 0.9 Hz), 7.78 (1H, s), 7.62 (1H, ddd, *J* = 8.3, 7.5, 0.9 Hz), 7.46 (1H, d, *J* = 8.3 Hz), 7.41 (1H, t, *J* = 7.5 Hz), 7.27 (1H, d, *J* = 8.0 Hz), 6.99 (1H, d, *J* = 8.0 Hz), 6.84 (1H, t, *J* = 8.0 Hz), 3.83 (3H, s).

### Synthesis of hydrazinyl thiazolyl coumarin derivatives 10g,h from (naphthalenylmethylene)thiosemicabazides 9a,b

The title compound was synthesized by a similar procedure described for the synthesis of **10a-f**, using 3-bromoacetylcoumarin (**4**) with naphthalenylmethylene)thiosemicabazide **9a,b**.

***(*E*)-3-{2-[2-(1-Naphthylidene)hydrazinyl]thiazol-4-yl}-2*H*-chromen-2-one (10g)***

The title compound (**10g**) (114 mg, 71%) was obtained after purification by recrystallization, using EtOAc–EtOH (3:1), as a yellow solid, mp 262–264°C (Lit. mp 262–265°C [20]; 240–242°C [15]); IR (KBr) ν_max_ 3424 (NH), 1702 (O-C = O), 1594 (C = N); ^1^H NMR (400 MHz, DMSO-*d*_*6*_) δ 12.35 (1H, br s), 8.79 (1H, d, *J* = 8.5 Hz), 8.71 (1H, s), 8.59 (1H, s), 8.01 (2H, t, *J* = 7.8 Hz), 7.88 (2H, d, *J* = 7.6 Hz), 7.83 (1H, s), 7.67 (1H, ddd, *J* = 8.1, 7.5, 0.9 Hz), 7.63 (1H, t, *J* = 8.5 Hz), 7.62 (2H, dd, *J* = 7.8, 1.8 Hz), 7.49 (1H, d, *J* = 8.1 Hz), 7.41 (1H, t, *J* = 7.5 Hz).

***(*E*)-3-{2-[2-(2-Hydroxynaphthylidene)hydrazinyl]thiazol-4-yl}-2H-chromen-2-one (10h)***

The title compound (10h) (119 mg, 79%) was obtained after purification by recrystallization, using EtOAc–EtOH (2:1), as a brown solid, mp 271–273°C, (Lit. mp 272–274°C [20]); IR (KBr) ν_max_ 3439 (NH), 3207 (NH), 1705 (O-C = O), 1602 (C = N); ^1^H NMR (400 MHz, DMSO-*d*_*6*_) δ 12.25 (1H, br s), 10.92 (1H, br s), 8.99 (1H, s), 8.78 (1H, d, *J* = 8.6 Hz), 8.59 (1H, s), 7.90-7.81 (3H, m), 7.82 (1H, s), 7.63 (1H, ddd, *J* = 8.2, 7.5, 1.5 Hz), 7.58 (1H, t, *J* = 7.5 Hz), 7.48 (1H, d, *J* = 8.2 Hz), 7.42 (2H, ddd, *J* = 8.2, 7.2, 4.0 Hz), 7.24 (1H, d, *J* = 8.4 Hz).

### Methods

^1^H and ^13^C NMR spectra were obtained using *d*_*6*_-dimethyl sulfoxide at 25°C using a Bruker DPX 400 instrument or 500 Avance instrument operating at 400 or 500 MHz for ^1^H spectra and 100 MHz for ^13^ C spectra, unless stated otherwise, and were reported in ppm; *J* values were recorded in Hz and multiplicities were expressed by the usual conventions (s = singlet, d = doublet, t = triplet, app = apparent, m = multiplet). Infra-red (IR) spectra were recorded in the range 4000–600 cm^-1^ on a Perkin-Elmer 1600 series FTIR spectrometer using KBr disks and are reported in cm^-1^. All compounds were examined by analytical thin layer chromatography carried out using aluminium-backed plates coated with Merck Kieselgel 60 GF_254_, eluting with hexane–ethyl acetate (3:1, *v/v*), that were visualised under UV light (at 254 and/or 360 nm). Microwave-assisted syntheses were carried out at the recorded temperature by the modulation of the initial magnetron power (given in parentheses) in a sealed tube using a CEM Discover single-mode instrument, with magnetic stirring and temperature measurement using the in-built IR sensor. Melting points (mp) were determined on a Kofler hot stage apparatus and are uncorrected. Commercially available reagents were used without further purification; solvents were dried by standard procedures.

### DPPH radical-scavenging activity

DPPH radical-scavenging activity of the samples was assessed by our reported method [43] with minor modifications. The 1,1-diphenyl-2-picrylhydrazyl radical solution was prepared by dissolving an appropriate amount of DPPH in MeOH to give a concentration of 1 mM. The DPPH radical solution (1 mM; 0.5 mL) was added to a solution of the compound to be tested in MeOH (4 mL) at various concentrations (to give a final concentration of DPPH of 0.11 mM). The mixture was shaken vigorously and incubated at room temperature in the dark for 30 min. The decrease in the absorbance of the resulting solution was then measured spectrophotometrically at *ν* 517 nm. All measurements were made in triplicate. Two controls were used for this test: a negative control (blank) consisting of MeOH (4 mL) and the DPPH radical solution (0.5 mL) and a positive control comprising the reference anti-oxidant (quercetin or BHT) in MeOH and DPPH radical solution. Inhibition of free radical DPPH in percentage was calculated as follows:

$$\text{Radical scavenging activity }\left(\%\right)=\left(A_{\text{blank}}-A_{\text{sample}}/A_{\text{blank}}\right)\times 100$$

Where *A*_*blank*_ is the absorbance of negative control (containing all reagents except test compounds) and *A*_*sample*_ is the absorbance of the test compounds and all the reagents. Sample concentration providing 50% inhibition (IC_50_) was calculated by plotting the inhibition percentage against sample concentration. All the synthesized compounds were evaluated for DPPH radical scavenging ability and the antioxidant activity of the synthesized compounds was compared with a synthetic antioxidant BHT (butylated hydroxytoluene) and a natural antioxidant quercetin, as the reference standards.

### ABTS^.+^ radical cation-scavenging activity

According to a modified method from Arnao et al. [45], a solution of ABTS (7.4 mM) in MeOH and a solution of potassium persulphate (2.6 mM) in MeOH were mixed in equal volumes and allowed to react for 12 hours in the dark at room temperature. The resulting solution (1.0 mL) was diluted (to a volume of 30.0 mL) by the addition of methanol to give an ABTS^·+^ concentration of 0.12 mM and absorbance of 1.1 ± 0.02 at 734 nm. A portion of the ABTS^·+^ solution (3.0 mL) was added to a methanolic solution (150 μL) of the compound to be tested at various concentrations and the resulting mixture was incubated in the dark for 2 hours. The absorbance of each solution was recorded at 734 nm. All measurements were made in triplicate and for each assay a fresh ABTS^·+^ stock solution was prepared. Radical scavenging activity was calculated in a similar fashion to the DPPH radical-scavenging assays. The IC_50_ value of each compound was calculated by plotting the inhibition percentage against concentration of the tested compounds and the results were expressed in μM.

## Competing interests

The authors declare that they have no competing interests.

## Authors’ contributions

HO and AA participated in study design and coordination, manuscript preparation and carried out the synthetic experiments, MCB and KCL participated in study design and coordination and manuscript preparation. All authors read and approved the final manuscript.

## Author details

^1^School of Chemical Sciences, Universiti Sains Malaysia, 11800, Penang, Malaysia. ^2^School of Pharmaceutical Sciences, Universiti Sains Malaysia, 11800, Penang, Malaysia. ^3^Department of Chemistry, School of Life Sciences, University of Sussex, Brighton BN1 9QJ, UK.
